# Biogeography of the Lizard Genus *Tropidurus* Wied-Neuwied, 1825 (Squamata: Tropiduridae): Distribution, Endemism, and Area Relationships in South America

**DOI:** 10.1371/journal.pone.0059736

**Published:** 2013-03-19

**Authors:** André Luiz Gomes de Carvalho, Marcelo Ribeiro de Britto, Daniel Silva Fernandes

**Affiliations:** 1 Richard Gilder Graduate School, American Museum of Natural History, New York, New York, United States of America; 2 Departamento de Vertebrados, Museu Nacional, Universidade Federal do Rio de Janeiro, Rio de Janeiro, Rio de Janeiro, Brazil; 3 Departamento de Zoologia, Universidade Federal do Rio de Janeiro, Rio de Janeiro, Rio de Janeiro, Brazil; University of Western Ontario, Canada

## Abstract

Based on comprehensive distributional records of the 23 species currently assigned to the lizard genus *Tropidurus*, we investigated patterns of endemism and area relationships in South America. Two biogeographic methods were applied, Parsimony Analysis of Endemicity (PAE) and Brooks Parsimony Analysis (BPA). Two areas of endemism were detected by PAE: the first within the domains of the semiarid Brazilian Caatinga, which includes seven endemic species, and the second in the region of the Serranía de Huanchaca, eastern Bolivia, in which three endemic species are present. The area cladograms recovered a close relationship between the Atlantic Forest and areas of the South American open corridor. The results revealed a close relationship among the provinces Caatinga (Cerrado, Parana Forest (Pantanal+Chaco)). The uplift of the Brazilian Central Plateau in the Late Pliocene-Early Pleistocene (4-2 Myr BP) has been interpreted as a major event responsible for isolation and differentiation of biotas along these areas. However, we emphasize that without the establishment of a temporal framework concerning the diversification history of *Tropidurus* it is premature to correlate cladogenetic events with specific time periods or putative vicariant scenarios. The limiting factors hampering the understanding of the biogeographic history of this genus include (1) the absence of temporal references in relation to the diversification of distinct clades within *Tropidurus*; (2) the lack of an appropriate taxonomic resolution of the species complexes currently represented by widely distributed forms; and (3) the need for a comprehensive phylogenetic hypothesis. We suggest that these three important aspects should be prioritized in future investigations.

## Introduction

*Tropidurus* Wied-Neuwied, 1825 is a large genus (23 known species) of South American lizards included in the family Tropiduridae whose species occupy open habitats of tropical and subtropical cis-Andean South America [1]–[5]. Despite the widespread distribution and local abundance of several species–accounting for large numbers of specimens preserved in scientific collections–no specific study focused on the biogeography of *Tropidurus* until the second half of the 1980s. Rodrigues [1]–[2] was the first author to produce distributional maps based on large museum samples and data obtained directly in the field. However, his main conclusions were established based on descriptive analyses of the morphological and distributional patterns observed. The first phylogenetic investigation focused on the internal relationships of *Tropidurus* was published only five years after Rodrigues has conducted his pioneering zoogeographic study [6]–[9]. Thus, all biogeographic hypotheses published in 1987 were decoupled from a phylogenetic context and remain opened to scrutiny.

Previous analyses of *Tropidurus* biogeography were greatly influenced by the Theory of Pleistocene Refuges [10]–[11], suggesting strict Quaternary scenarios to explain the diversification history and distribution of the genus [1]–[2]. This biogeographic paradigm states that continuously forested areas became isolated nuclei due to the occurence of the glacial cycles. Similarly, during phases of climate relaxation (*i.e.* interglacial periods), savannas were reduced to isolated nuclei amid the ingrown forests in expansion. Within these nuclei, events of allopatric speciation were hypothesized to have occurred in response to geographic and genetic isolation [10]. These large-scale landscape changes are assumed to explain numerous savanna relicts enclaved in the forested domains of South America, as well as isolated forested refugia enclosed by open savanna landscapes [10], [12]–[13]. The origin and distribution of taxa that inhabit (or inhabited) areas affected by Quaternary glacial cycles were promptly associated with isolation in refugia [14]–[17], and the evolutionary history and distributional patterns of *Tropidurus* were similarly interpreted as outcomes of these events [1]–[2].

Although the impacts of the Quaternary glacial cycles on the biodiversity of South America are undeniable [18]–[24], we should not assume that *Tropidurus* species originated during this period simply because their distributions match areas that underwent landscape changes during glacial cycles. A growing number of studies carried out with the aid of molecular tools and paleontological evidence have pointed out that the origin of many South American vertebrate genera and species occurred during the Tertiary [25]–[27]. Antonelli *et al.* [28], for example, conducted a comprehensive review of molecular and phylogeographic studies of several groups of tetrapods in the Amazon region and achieved the same conclusion for most of them (*i.e.* diversification occurring in the Miocene-Pliocene), emphasizing an overvaluation of the Quaternary in the South American biogeographic scenario.

Nevertheless, results obtained through the analysis of molecular clocks offer only an initial step towards understanding the diversification events and evolution of species distributions. The identification of vicariant processes accounting for diversification depends on analyses concentrated on recovering patterns of area breakups and endemism. Despite all previously published evidence, we tend to adhere to the idea that the Quaternary represented the culmination of a long history of diversification in South America and its strongest impacts do not explain the origin of most taxa, but the re-arrangement of their distributions. However, basic questions concerning the biogeographic history of South America remain unresolved: (1) Where are the areas of endemism located and how much diversity do they comprise? (2) How congruent are the distributional and phylogenetic patterns among different taxa occupying such areas? (3) What are the contributions of geological and climatological events to vicariance scenarios? (4) How did the vicariant events shape the diversification of biological groups with different dispersion abilities? (5) Is the biogeographic history of South America marked by regular, random, or explosive processes affecting distributions and diversity?

Cracraft [29] was the first to perform a global biogeographic analysis on a continental scale for South America, where 33 areas of endemism were detected based on the distributional congruence of a large number of avian groups. Porzecanski and Cracraft [30] reanalyzed that database, along with data of Haffer [31], and hypothesized that the patterns of area relationships recovered were associated with Tertiary vicariant events. In that same year, employing a compilation of the composition of 32 Central and South American lizard communities, Colli [32] proposed that the most profound divergences in the lineages of the South American herpetofauna were established in the Late Cretaceous, a period in which the fundamental dichotomy between humid and hot *versus* xeric and cold regions had already been established in the continent. However, Colli argued that the Tertiary was the period in which the modern biota was effectively defined. This is a hypothesis under growing acceptance (see review in Ref. [33]).

The biogeographic hypotheses established by Porzecanski and Cracraft [30] and Colli [32] are significantly congruent, but both studies adopted analyses without an intrinsic phylogenetic component. Although the employment of different taxonomic levels in the Cladistic Analysis of Distributions and Endemism (CADE) is argued to incorporate phylogenetic information of the taxa to recover historical patterns of area relationships, it does not truly incorporate phylogeny because it does not depart from taxon cladograms to generate area cladograms. Biogeographic analyses based on the distribution of South American monophyletic groups represent, in turn, operative tests directed to corroborate or refute area relationship hypotheses. Hence, to detect areas of endemism and test the hypothesis of close historical relationships among areas composing the South American open corridor, we performed a cladistic biogeographic analysis based on the distributional records of the lizard genus *Tropidurus* using Brooks Parsimony Analysis (BPA) and Parsimony Analysis of Endemicity (PAE). Our study addresses four major questions: (1) How many areas of endemism can be recovered based on the distribution of *Tropidurus*? (2) What are the patterns of area relationships recovered based on the distributional and phylogenetic information compiled for *Tropidurus*? (3) Are the patterns of area relationships in agreement with previously published hypotheses (*e.g.* (Brazilian Atlantic Forest (Caatinga (Cerrado+Chaco))? (4) Is it possible to identify putative vicariant events associated with the patterns of area relationships and species distribution analyzed?

## Materials and Methods

### Data Collection and Preparation of Maps

All analyses employed the distributional dataset compiled by the senior author [5]. This dataset was produced based on an exhaustive survey of the literature and collection records of the most representative Brazilian museums for *Tropidurus*, accessed to review specimen records and identifications. The accessed museums were: Museu Nacional, Universidade Federal do Rio de Janeiro (MNRJ), Rio de Janeiro, RJ; Museu de Zoologia, Universidade de São Paulo (MZUSP), São Paulo, SP; Coleção Herpetológica da Universidade de Brasília (CHUNB), Brasília, DF; Coleção Herpetológica da Universidade Federal Rural do Rio de Janeiro (RU), Seropédica, RJ; Museu Paraense Emilio Goeldi (MPEG), Belém, PA; Museu de Zoologia da Universidade Estadual de Santa Cruz (MZUESC), Santa Cruz, BA; Coleção Herpetológica da Comissão Executiva do Plano da Lavoura Cacaueira (CEPLAC), Ilhéus, BA; and Instituto Nacional de Pesquisas da Amazônia (INPA), Manaus, AM. Geographic coordinates associated with specimen records housed in zoological collections were preferentially considered. Localities without geographic coordinates in the original source were georeferenced online with aid of gazetteers or using Google Earth version 6.1 [34]. Museum records devoid of geographic coordinates and mentioning exclusively names of provinces or states were not considered to avoid inaccuracy. All geographic coordinates were converted to decimal degrees and subsequently imported into the program Arc GIS version 10.1 [35], where distributional maps used for biogeographic analyses were produced.

### Parsimony Analysis of Endemicity

PAE [36]–[37] is a method of historical biogeography used to recover natural distribution patterns of organisms [38]–[39]. The method is based on the assumption zero [40] and considers the shared occurrences of species among areas as evidence of common history [36]–[39]. PAE employs presence/absence data to recover relationships based on two underlying assumptions: (1) the absence of a taxon is “primitive” and its presence is “derived”, and (2) the hypothetical “ancestral” or “outgroup” area is one in which none of the sample sets of the current taxa exist. Although based on a cladistic methodology, PAE is not a cladistic method because it does not depart from the phylogeny of the taxa to construct area cladograms [36]–[39]. Because it disregards the phylogenetic relationships of the species and considers vicariance as the main process responsible for determining biogeographic patterns, the method has been subject of intense criticism [41]–[44]. However, congruent results in relation to those generated by cladistic methods have been obtained, which suggests that natural historical patterns are recovered by PAE in several cases [43].

In addition to the investigation of patterns of area relationships, PAE is a biogeographical method able to detect areas of endemism [45]. These areas represent hypotheses of natural entities essentially adopted as operational geographic units during historical biogeographic reconstructions [45]–[48]. Areas of endemism originate through the fragmentation of an ancestral biota by the appearance of a geographic barrier that promotes spatially concordant events of allopatric speciation in different groups of organisms, responsible for the emergence of two new biotas [48]–[54]. Similar responses of different taxa to the same vicariant event generate similar phylogenetic patterns. It is then expected that organisms composing the same biota, subjected to the same vicariant events, display congruent phylogenetic patterns [49]–[52], [55]–[56]. Therefore, through the analysis of the levels of distributional and phylogenetic congruence among different taxa, it is possible to reconstruct the history of diversification (in a spatial and temporal perspective) of the areas occupied by these organisms.

**Figure 1:**
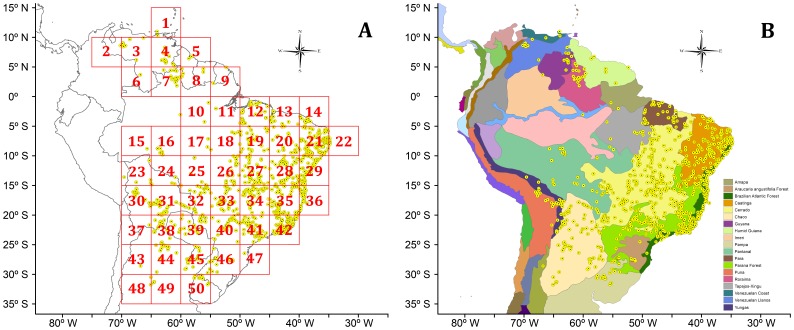
Distributional maps of the lizard genus *Tropidurus* showing (A) the 5°×5° quadrats employed as operational geographic units by PAE and (B) the biogeographic provinces of South America [–[59] employed as operational geographic units by PAE and BPA. Yellow dots represent collection points of *Tropidurus* confirmed through the analysis of voucher specimens and literature (for additional information see Ref. [5]).

Analyzing the distribution of the lizard genus *Tropidurus*, PAE was implemented to detect areas of endemism and to identify patterns of area relationships in South America. We adopted the protocols proposed by Morrone [45], employing 5°×5° quadrats and the biogeographic provinces of South America defined by Morrone [57]–[58] as operational geographic units (Fig. 1A). To obtain a single area cladogram, we applied majority rule consensus [59] (50% cut-off) to the set of most parsimonious trees recovered by PAE. Although this cladogram does not represent the direct result of the parsimony analysis (*i.e.* the fundamental cladograms), this is the only way to summarize the common components between fundamental cladograms. The optimization of character states (*i.e.* species presence/absence) was performed on the consensus cladogram. Only areas supported by the presence of two or more taxa with exclusive distribution were considered for identifying areas of endemism [45].

### Brooks Parsimony Analysis

BPA was adopted with the purpose of reconstructing patterns of area relationships in South America and evaluating the results initially established by PAE; this time employing the phylogenetic information as additional evidence for the reconstruction of the historical relationships. Brooks [60]–[62] originally proposed the employment of parsimony analysis for the study of co-speciation between parasites and hosts. Employing the phylogeny of parasites and their patterns of association to the hosts, the method proved to be able to properly reconstruct the phylogeny of the latter. BPA is based on the idea that a parasite species can be associated with a host as a result of two distinct events: (1) the ancestor of the parasite species was associated with the ancestor of its host, resulting in association by descent, or (2) the parasite species evolved with a host, moving later to another, resulting in an association by colonization [62]. Therefore, parasites can be interpreted as characters that can be subjected to cladistic examination for reconstruction of the historical relationships among host species, as it would be possible with the use of other sources of characters (*e.g.* morphological, physiological, behavioral, molecular, etc.) [60]–[62].

**Figure 2:**
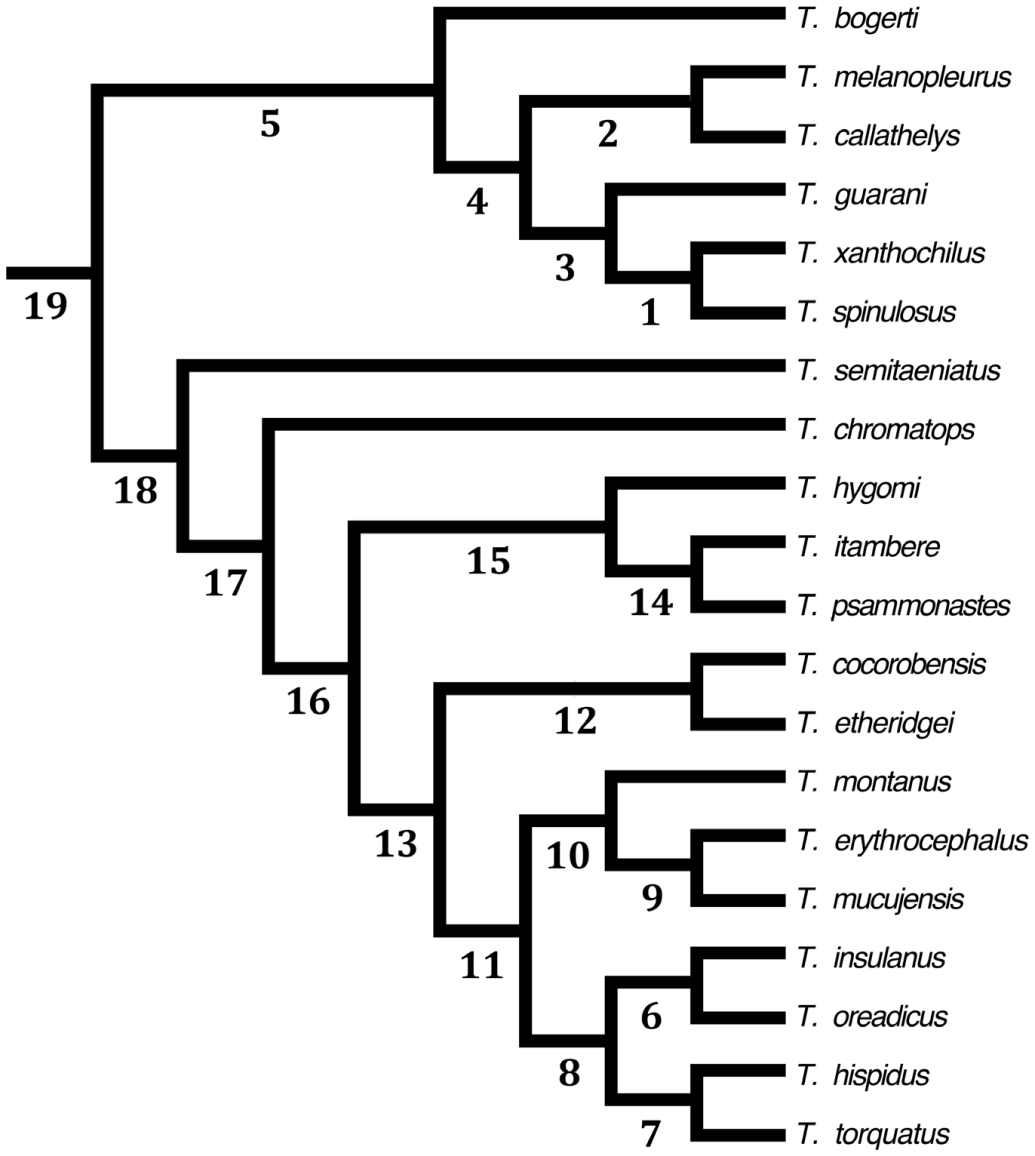
Phylogenetic relationships of *Tropidurus* (adapted from Frost *et al.* [**9**] **) with hypothetical ancestors (represented by numbers 1–19) defined for implementation of BPA (see also** **Table 3** **).**

The model proposed by Brooks for the reconstruction of the co-evolutionary relationships between parasites and hosts can be effectively applied to biogeographic contexts [63]–[65]. In this new approach, distribution areas were considered analogous to the hosts and the taxa occupying these areas considered analogous to the parasite species. Associations by descent are understood as a direct result of vicariant events responsible for cladogenesis, while associations by colonization represent colonization events (*i.e*. dispersions) between areas [63]–[65]. BPA was revised over the past decades, with most adaptations concerning character coding and the implementation of area duplication as a strategy to deal with hypothesized reticulations and inconsistencies introduced by widespread species, redundant distributions or absences (*e.g.* Ref. [63]–[67]). Tests have shown a great sensitivity of this method to investigation of a broad range of biogeographic events, highlighting its ability to deal with noise resulting from dispersion events or non-response to vicariance [63]–[64], [68]. Because only one group of organisms is analyzed, BPA was employed following its original proposition [60], [67], based on the phylogenetic relationships of the lizard genus *Tropidurus* proposed by Frost *et al.* [9] (Fig. 2). To allow comparisons with PAE, the biogeographic provinces defined by Morrone [57]–[58] were adopted as operational geographic units (Fig. 1B). Similarly, in order to obtain a single area cladogram, majority rule consensus [59] (50% cut-off) was implemented. Following the same procedures applied to PAE, the optimizations of character states were performed on the consensus cladogram.

### Programs and Algorithms

Tree searches were carried out in TNT version 3.1 [69]. Traditional heuristic searches were based on 100 replicates and 10,000 trees were saved per replicate, using the stepwise addition algorithm and rearrangement of branches through tree bisection-reconnection [70]. All analyses were repeated using new technologies to improve the exploration of tree space and to guarantee the robustness of the results previously found using TBR. Sectorial search [71], ratchet [72], and tree fusing [71] were associated under driven search, with initial addseqs  = 10, until the best scoring tree was found 100,000 times.

## Results and Discussion

### Distribution

*Tropidurus* is distributed over open habitats of the tropical and subtropical cis-Andean South America. The species occur predominantly along the South American arid diagonal formed by the biomes Caatinga, Cerrado, and Chaco, in enclaves of savanna formations in Amazonia, and in a large area of the Brazilian Atlantic coast [1]–[5]. Species distributions range from Venezuela (and one locality by the Colombian border), Guyana, Suriname, and French Guiana in the north, to northern Argentina and Uruguay in the south. *Tropidurus* occupies a vast area of Brazil, extending west to Bolivia and Paraguay. The entire distribution comprises territories belonging to 11 countries and 20 biogeographic provinces, placing *Tropidurus* as one of the most widely distributed lizard genera of South America [5] (Fig. 1).

Comparative analyses of *Tropidurus* distributions showed that phylogenetically closely related species have distinct distributional patterns and range sizes. However, the occurrence of either widely distributed or locally distributed species in the genus is noticeable [2], [5]. The dichotomous association with rock outcrops or sandy soils is a determinant component shaping species distribution in a local and geographical scale [1]–[2], [73]–[74]. In addition, forested environments act as extremely efficient barriers for these typically heliothermic lizards and several cases of disjunct or isolated distributions are limited by humid forested habitats. Carvalho [5] provided a detailed analysis of *Tropidurus* distribution, including distribution maps for all species and critical comments covering taxonomic, biogeographic, and conservation issues. For comprehensive descriptions of distributional patterns and updated distribution maps, refer to that publication.

### Areas of Endemism

**Figure 3:**
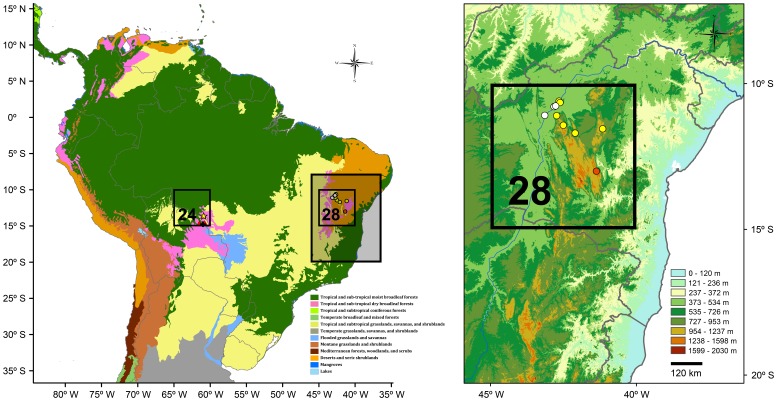
Areas of endemism detected by PAE (left map) based on the distribution of the lizard genus *Tropidurus* employing 5°×5° quadrats as operational geographic units [**45**]. The area of endemism located within the Quadrat 24 (enlarged in Figure 4) comprises the Noel Kempf Mercado National Park (including the Serranía de Huanchaca) and El Refugio Biological Station, in the Department of Santa Cruz, eastern Bolivia, and was supported by the occurrence of *T. callathelys* (yellow star), *T. chromatops* (red triangles), and *T. xanthochilus* (black cross). The area of endemism located within the Quadrat 28 (enlarged) comprises the southern portion of the Caatinga province, northeastern Brazil, and was supported by the endemics *T. erythrocephalus* (yellow dots), *T. mucujensis* (orange dot), and *T. psammonastes* (white dots).

PAE based on quadrats identified two areas of endemism in South America, circumscribed by quadrats 28 and 24 (Fig. 3, Table 1). The first area is located within the domains of the semiarid Brazilian Caatinga and found support on the occurrence of three endemic species: *T. erythrocephalus*, *T. mucujensis*, and *T. psammonastes*. This province comprises the largest nucleus of seasonally dry tropical forest in the Neotropics [75]–[76], but deserves special attention as one of the most threatened environments of South America [33], [77]. The second area of endemism is included in a diverse transitional zone located in eastern Bolivia, marked by the contact of distinct savanna formations, the humid amazon forest, and patches of seasonally dry tropical forests [78]. This area was supported by the presence of three endemic species: *T. callathelys*, *T. chromatops*, and *T. xanthochilus*.

**Table 1:** Data matrix of the 23 *Tropidurus* species used in the Parsimony Analysis of Endemicity (PAE) employing quadrats as operational geographic units [45].

Species	01	02	03	04	05	06	07	08	09	10	11	12	13	14	15	16	17	18	18	20	21	22	23
Outgroup	0	0	0	0	0	0	0	0	0	0	0	0	0	0	0	0	0	0	0	0	0	0	0
Quadrat 1	0	0	0	0	0	0	0	0	1	0	0	0	0	0	0	0	0	0	0	0	0	0	0
Quadrat 2	0	0	0	0	0	0	0	0	1	0	0	0	0	0	0	0	0	0	0	0	0	0	0
Quadrat 3	0	0	0	0	0	0	0	0	1	0	0	0	0	0	0	0	0	0	0	0	0	0	0
Quadrat 4	1	0	0	0	0	0	0	0	1	0	0	0	0	0	0	0	0	0	0	0	0	0	0
Quadrat 5	0	0	0	0	0	0	0	0	1	0	0	0	0	0	0	0	0	0	0	0	0	0	0
Quadrat 6	0	0	0	0	0	0	0	0	1	0	0	0	0	0	0	0	0	0	0	0	0	0	0
Quadrat 7	0	0	0	0	0	0	0	0	1	0	0	0	0	0	0	0	0	0	0	0	0	0	0
Quadrat 8	0	0	0	0	0	0	0	0	1	0	0	0	0	0	0	0	0	0	0	0	0	0	0
Quadrat 9	0	0	0	0	0	0	0	0	1	0	0	0	0	0	0	0	0	0	0	0	0	0	0
Quadrat 10	0	0	0	0	0	0	0	0	1	0	1	0	0	0	0	0	0	0	0	0	0	0	0
Quadrat 11	0	0	0	0	0	0	0	0	1	0	0	0	0	0	0	0	0	0	0	0	0	0	0
Quadrat 12	0	0	0	0	0	0	0	0	1	0	0	0	0	0	0	0	1	0	0	0	0	0	0
Quadrat 13	0	0	0	0	0	0	0	0	1	0	0	0	0	0	0	0	0	0	0	1	0	0	0
Quadrat 14	0	0	0	0	0	0	0	0	1	0	0	0	0	0	0	0	0	0	0	1	0	0	0
Quadrat 15	0	0	0	0	0	0	0	0	0	0	0	0	0	0	0	0	1	0	0	0	0	0	0
Quadrat 16	0	0	0	0	0	0	0	0	0	0	0	0	0	0	0	0	1	0	0	0	0	0	0
Quadrat 17	0	0	0	0	0	0	0	0	0	0	1	0	0	0	0	0	0	0	0	0	0	0	0
Quadrat 18	0	0	0	0	0	0	0	0	0	0	0	0	0	0	0	0	1	0	0	0	0	0	0
Quadrat 19	0	0	0	0	0	0	0	0	1	0	0	0	0	0	0	0	1	0	0	1	0	1	0
Quadrat 20	0	0	0	0	0	0	0	1	1	0	0	0	0	0	0	0	0	1	0	1	0	0	0
Quadrat 21	0	0	0	1	0	0	0	0	1	0	0	0	1	0	0	0	0	0	0	1	0	0	0
Quadrat 22	0	0	0	0	0	0	0	0	1	0	0	0	0	0	0	0	0	0	0	0	0	0	0
Quadrat 23	0	0	0	0	0	1	0	0	0	0	0	0	0	1	0	0	1	0	0	0	0	0	0
Quadrat 24	0	1	1	0	0	1	0	0	0	0	0	0	0	0	0	0	0	0	0	0	1	0	1
Quadrat 25	0	0	0	0	0	1	1	0	0	0	0	1	0	0	0	0	0	0	0	0	0	1	0
Quadrat 26	0	0	0	0	0	0	0	0	0	0	1	1	0	0	0	0	1	0	0	0	0	1	0
Quadrat 27	0	0	0	0	0	1	0	0	1	0	0	1	0	0	0	0	1	0	0	0	0	1	0
Quadrat 28	0	0	0	1	1	1	0	0	1	0	0	0	0	0	1	1	1	1	1	1	0	0	0
Quadrat 29	0	0	0	0	0	0	0	0	1	1	0	0	0	0	0	0	0	0	0	1	0	1	0
Quadrat 30	0	0	0	0	0	1	0	0	0	0	0	0	0	1	0	0	0	0	0	0	0	0	0
Quadrat 31	0	0	0	0	0	1	0	0	0	0	0	0	0	1	0	0	0	0	0	0	1	0	0
Quadrat 32	0	0	0	0	0	1	1	0	0	0	0	1	0	0	0	0	1	0	0	0	1	1	0
Quadrat 33	0	0	0	0	0	1	1	0	0	0	0	1	0	0	0	0	1	0	0	0	0	1	0
Quadrat 34	0	0	0	0	0	1	0	0	0	0	0	1	0	0	0	0	1	0	0	0	0	1	0
Quadrat 35	0	0	0	0	0	1	0	0	1	0	0	0	0	0	1	0	1	0	0	0	0	1	0
Quadrat 36	0	0	0	0	0	0	0	0	0	0	0	0	0	0	0	0	0	0	0	0	0	1	0
Quadrat 37	0	0	0	0	0	0	0	0	0	0	0	0	0	1	0	0	0	0	0	0	0	0	0
Quadrat 38	0	0	0	0	0	1	0	0	0	0	0	0	0	1	0	0	0	0	0	0	1	0	0
Quadrat 39	0	0	0	0	0	0	1	0	0	0	0	1	0	0	0	0	1	0	0	0	1	1	0
Quadrat 40	0	0	0	0	0	0	1	0	0	0	0	1	0	0	0	0	0	0	0	0	0	1	0
Quadrat 41	0	0	0	0	0	0	0	0	0	0	0	1	0	0	0	0	0	0	0	0	0	1	0
Quadrat 42	0	0	0	0	0	0	0	0	1	0	0	1	0	0	1	0	0	0	0	0	0	1	0
Quadrat 43	0	0	0	0	0	0	0	0	0	0	0	0	0	0	0	0	0	0	0	0	1	0	0
Quadrat 44	0	0	0	0	0	1	0	0	0	0	0	0	0	0	0	0	0	0	0	0	1	0	0
Quadrat 45	0	0	0	0	0	1	1	0	0	0	0	0	0	0	0	0	0	0	0	0	0	1	0
Quadrat 46	0	0	0	0	0	0	0	0	0	0	0	0	0	0	0	0	0	0	0	0	0	1	0
Quadrat 47	0	0	0	0	0	0	0	0	0	0	0	0	0	0	0	0	0	0	0	0	0	1	0
Quadrat 48	0	0	0	0	0	1	0	0	0	0	0	0	0	0	0	0	0	0	0	0	0	0	0
Quadrat 49	0	0	0	0	0	0	0	0	0	0	0	0	0	0	0	0	0	0	0	0	1	0	0
Quadrat 50	0	0	0	0	0	0	0	0	0	0	0	0	0	0	0	0	0	0	0	0	0	1	0

Absence of taxon codified as “0”, presence codified as “1”. Taxa: (1) *T. bogerti*, (2) *T. callathelys*, (3) *T. chromatops*, (4) *T. cocorobensis*, (5) *T. erythrocephalus*, (6) *T. etheridgei*, (7) *T. guarani*, (8) *T. helenae*, (9) *T. hispidus*, (10) *T. hygomi*, (11) *T. insulanus*, (12) *T. itambere*, (13) *T. jaguaribanus*, (14) *T. melanopleurus*, (15) *T. montanus*, (16) *T. mucujensis*, (17) *T. oreadicus*, (18) *T. pinima*, (19) *T. psammonastes*, (20) *T. semitaeniatus*, (21) *T. spinulosus*, (22) *T. torquatus*, (23) *T. xanthochilus*.

**Figure 4:**
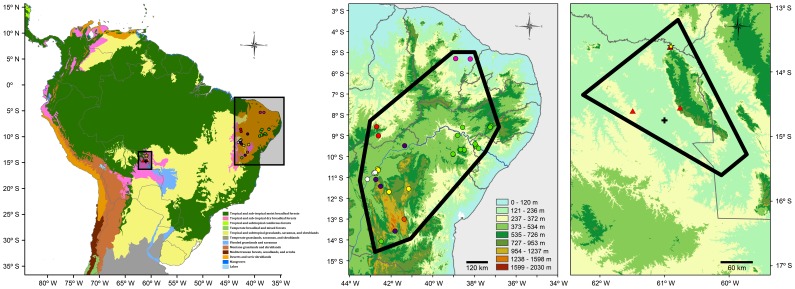
Areas of endemism detected by PAE (left map) based on the distribution of the lizard genus *Tropidurus* employing the biogeographic provinces of South America [**58]**–[**59**] as operational geographic units. The area of endemism located in eastern Bolivia (enlarged, right map) corresponds to that circumscribed by Quadrat 24 in Figure 3, comprising the Noel Kempf Mercado National Park (including the Serranía de Huanchaca) and El Refugio Biological Station, in the Department of Santa Cruz, and was supported by the occurrence of the same endemic species, *T. callathelys* (yellow star), *T. chromatops* (red triangles), and *T. xanthochilus* (black cross). The area of endemism detected in northeastern Brazil (enlarged, center map) is located within the Caatinga province and was supported by the endemics *T. cocorobensis* (green dots), *T. erythrocephalus* (yellow dots), *T. helenae* (red dots), *T. jaguaribanus* (pink dots), *T. mucujensis* (orange dot), *T. pinima* (purple dots), and *T. psammonastes* (white dots). Altitudinal legend corresponds to center and right enlarged maps.

PAE based on the biogeographic provinces of South America detected two areas of endemism (Fig. 4, Table 2). The area located in the Pantanal province coincided with that circumscribed by Quadrat 24 and found support on the occurrence of the same endemic species. However, the second area of endemism detected is represented by the Caatinga province and supported by the occurrence of seven endemic species. The list includes the three species restricted to Quadrat 22 and four additional endemics: *T. cocorobensis*, *T. jaguaribanus, T. helenae*, and *T. pinima*. The area of endemism identified here is geographically larger and richer in endemics than previously recognized, which demonstrates the impact of the delimitation of the operational geographic units on the results recovered by PAE. This result is in accordance with previous studies showing that areas that more accurately represent natural units (*e.g.* ecorregions, provinces) significantly improve area cladogram resolution and endemism detection compared to the use of quadrats [79].

**Table 2:** Data matrix of the 23 *Tropidurus* species used in the Parsimony Analysis of Endemicity (PAE) employing the biogeographic provinces of South America as operational geographic units [57]–[58].

Species	01	02	03	04	05	06	07	08	09	10	11	12	13	14	15	16	17	18	19	20	21	22	23
Outgroup	0	0	0	0	0	0	0	0	0	0	0	0	0	0	0	0	0	0	0	0	0	0	0
Venezuelan Coast	0	0	0	0	0	0	0	0	1	0	0	0	0	0	0	0	0	0	0	0	0	0	0
Venezuelan Llanos	0	0	0	0	0	0	0	0	1	0	0	0	0	0	0	0	0	0	0	0	0	0	0
Imeri	0	0	0	0	0	0	0	0	1	0	0	0	0	0	0	0	0	0	0	0	0	0	0
Guyana	1	0	0	0	0	0	0	0	1	0	0	0	0	0	0	0	0	0	0	0	0	0	0
Humid Guyana	0	0	0	0	0	0	0	0	1	0	0	0	0	0	0	0	0	0	0	0	0	0	0
Roraima	0	0	0	0	0	0	0	0	1	0	0	0	0	0	0	0	0	0	0	0	0	0	0
Amapa	0	0	0	0	0	0	0	0	1	0	0	0	0	0	0	0	0	0	0	0	0	0	0
Tapajos-Xingu	0	0	0	0	0	0	0	0	1	0	1	0	0	0	0	0	1	0	0	0	0	0	0
Para	0	0	0	0	0	0	0	0	1	0	0	0	0	0	0	0	1	0	0	1	0	0	0
Pantanal	0	1	1	0	0	1	1	0	0	0	1	1	0	0	0	0	1	0	0	0	1	1	1
Yungas	0	0	0	0	0	1	0	0	0	0	0	0	0	1	0	0	0	0	0	0	0	0	0
Caatinga	0	0	0	1	1	1	0	1	1	1	0	0	1	0	1	1	0	1	1	1	0	0	0
Cerrado	0	0	0	0	0	1	1	0	1	0	1	1	0	0	1	0	1	0	0	1	0	1	0
Chaco	0	0	0	0	0	1	1	0	0	0	0	0	0	1	0	0	0	0	0	0	1	1	0
Pampa	0	0	0	0	0	0	0	0	0	0	0	0	0	0	0	0	0	0	0	0	0	1	0
Monte	0	0	0	0	0	0	0	0	0	0	0	0	0	0	0	0	0	0	0	0	1	0	0
Brazilian Atlantic Forest	0	0	0	0	0	0	0	0	1	1	0	1	0	0	0	0	0	0	0	1	0	1	0
Parana Forest	0	0	0	0	0	1	1	0	1	0	0	1	0	0	1	0	0	0	0	1	0	1	0
*Araucaria angustifolia* Forest	0	0	0	0	0	0	0	0	0	0	0	1	0	0	0	0	0	0	0	0	0	1	0
Puna	0	0	0	0	0	1	0	0	0	0	0	0	0	1	0	0	0	0	0	0	0	0	0

Absence of taxon codified as “0”, presence codified as “1”. Taxa: (1) *T. bogerti*, (2) *T. callathelys*, (3) *T. chromatops*, (4) *T. cocorobensis*, (5) *T. erythrocephalus*, (6) *T. etheridgei*, (7) *T. guarani*, (8) *T. helenae*, (9) *T. hispidus*, (10) *T. hygomi*, (11) *T. insulanus*, (12) *T. itambere*, (13) *T. jaguaribanus*, (14) *T. melanopleurus*, (15) *T. montanus*, (16) *T. mucujensis*, (17) *T. oreadicus*, (18) *T. pinima*, (19) *T. psammonastes*, (20) *T. semitaeniatus*, (21) *T. spinulosus*, (22) *T. torquatus*, (23) *T. xanthochilus*.

Until the 1980s the Caatinga was considered a poor and depleted area with respect to its vertebrate fauna [80]–[84]. However, several works have gradually identified endemic taxa associated with xeric formations and humid forest relicts [29], [22], [85]–[88]. The detection of seven *Tropidurus* species endemic to the Caatinga constitutes additional evidence against the alleged lack of faunal identity of this biome, supporting its position as the largest, most isolated and species-rich Neotropical nucleus of seasonally dry tropical forests [77], [89]. The area of endemism detected in the Caatinga comprises, for instance, the northern portion of the Espinhaço mountain range (Chapada Diamantina Complex) and the interior sand dune fields of the São Francisco River, two exceptionally diverse areas [85]–[87]. Although these and other regions were found to be part of a single area of endemism, floristic and structural evidence suggest that they represent discrete historical units with biotic and abiotic attributes that shape species distribution and endemism within the Caatinga [33], [90]. Thus, we acknowledge that the identification of multiple or nested areas of endemism within this province will likely emerge from regional scale analyses focused on the detection of congruent spatial and phylogenetic patterns comprising distinct biological groups.

Two *Tropidurus* species endemic to the Caatinga, *T. cocorobensis* and *T. psammonastes*, have distributions defined by the occurrence of sandy soils. This pattern is consistent with the results found by Queiroz [91] for leguminous plants, demonstrating the existence of two distinct biotas within the Caatinga: one associated with soils derived from crystalline surfaces and another with emerged sedimentary surfaces. Queiroz [91] suggested that sandy areas harbor the majority of the flora endemic to the Caatinga and that these areas were partially replaced during the Late Tertiary and Early Quaternary when geological pediplanation exposed the crystalline surfaces. The evolutionary history of these biotas could be investigated through the combination of molecular studies estimating the diversification ages of the two major floristic divisions and phylogeographic analyses estimating timing and routes of expansion of the associated taxa [33]. These data are not currently available for *Tropidurus*, and to our knowledge for any other vertebrate group within the Caatinga.

Werneck [33] highlighted that the largest part of the herpetofauna endemic to the Caatinga is associated with sandy soils (which are suggested to have been much more widely distributed in the past [85], [92]), and pointed out that although the sand dunes of Rio São Francisco cover only about 0.8% of the total Caatinga area, they comprise 27% of the squamate fauna endemic to the province [85], [91]. One component of the remarkable lizard diversity of this area is *Tropidurus psammonastes*, a psammophilous species with distribution restricted to the sand dune banks of the left margin of the São Francisco River [93]. Rodrigues [85], [94]–[95] proposed an allopatric speciation hypothesis entitled paleolacustrine hypothesis to explain the origin and distribution of several pairs of phylogenetically related species living in opposite banks of the river. The model suggests that after the Wisconsin-Würm glaciation, about 12,000 years BP, the São Francisco River started cutting through the sand dune fields formed by erosion of the quartizitic boulder of the Espinhaço mountain range and changed its drainage pattern to exorheic, draining into the Atlantic coast. This event was allegedly responsible for the isolation of populations in sand dune banks located on opposite margins of the river, preventing gene flow and promoting speciation.

The São Francisco River is one of the largest river systems in Brazil and potentially represents an effective geographical barrier. However, the model proposed by Rodrigues [85], [94]–[95] suggests extremely recent events as responsible for the endemic species occupying the sand dune fields. Passoni *et al.* [96] conducted a molecular study of the tropidurine lizard genus *Eurolophosaurus*, which has two of its three species restricted to sandbanks of the São Francisco River, and revealed that the period of divergence between species inhabiting these areas ranges from 5.4–1.5 Myr BP, exceeding considerably the 12,000 years previously hypothesized. Siedchlag *et al.* [97] provided additional data supporting a late Miocene-Pliocene vicariant history of two genera of spectacled lizards, *Calyptomatus* Rodrigues, 1991 (with divergence of phylogenetically related species occupying opposite banks of the São Francisco River between 6.5–1.8 Myr BP) and *Nothobachia* Rodrigues, 1984 (with divergence of phylogenetically related populations occupying opposite banks of the São Francisco River between 3.0–4.0 Myr BP). Although the period of isolation and divergence of *T. psammonastes* is unknown, the results previously found for other lizard groups endemic to the sand dunes banks of the São Francisco River demonstrate that the biota of the region has a diversification history dating back to the Tertiary. Hence, *T. psammonastes* is not expected to be an exception.

The endemic *T. erythrocephalus* and *T. mucujensis* are restricted to the high-altitude savanna-like environments known as campos rupestres [1], [5], which cover litholic soils above 900 m along the Espinhaço mountain range [98]. These species form the sister clade to *T. montanus*, composing a group whose evolution is hypothesized to have occured in strict association with *campos rupestres*. The Espinhaço is geographically included within the boundaries of the Cerrado (central and southern portion) and Caatinga (northern portion), however the high number of species and genera endemic to *campos ruspestres* along this mountain range [98]–[103] indicates that these formations possibly represent a biological unit with a long history of independent evolution [104]. The distribution of the endemic *Tropidurus* species in different sections of the Espinhaço shows that geographical factors might have been prevalent throughout their evolutionary history. These allopatric ranges support recent analyses demonstrating that different sections of *campos rupestres* comprise distinct areas of endemism [105]. Future proposals of regionalization should consider these areas with special attention and avoid the equivocal association of *campos rupestres* endemics to neighbouring historically unrelated provinces [104].

The four species in the *T. semitaeniatus* group occur in the area of endemism located in the Caatinga, but only three of them (*T. helenae*, *T. jaguaribanus*, and *T. pinima*) were detected as endemic elements. These species have features adapted to life in crevices between rock blocks (including expressive dorsovental body flattening, cryptic coloration, and reduced number of elongated eggs) [106] and occupy rock outcrops scattered throughout the area [107]–[111]. The highly adapted morphology and tight association with rock outcrops suggest that the patchy distribution of these environments (in some cases surrounded by extensive areas of sandy soils [85], [109]) affects the distributional range of these lizards. Two lines of evidence support a vicariant diversification history within the *T. semitaeniatus* species group: (1) the restricted and allopatric distributions of the species (with exception of *T. semitaeniatus*, but see next topic), and (2) the geographically structured molecular variation among populations of the widely distributed *T. semitaeniatus* (M.T. Rodrigues, pers. comm.), indicating that several species remain to be diagnosed and described. In accordance with the historical climate stability hypothesis–which states that stable areas permit more species to arise and persist, resulting in high species diversity and endemism [112]–[114]–the occurrence of a high number of endemic species within the monophyletic *T. semitaeniatus* group might corroborate the existence of the long-term climatic stability refugium identified within the Caatinga [77]. However, the effective processes behind the diversification events remain unknown, although the direct observation of the distribution patterns suggests that allopatric speciation models resulting from isolation in plateaus and valleys deserve examination.

The second area of endemism detected by PAE comprises the Serranía de Huanchaca and adjacent areas at the Noel Kempf Mercado National Park and El Refugio Biological Station, in the Department of Santa Cruz, eastern Bolivia. The area is divided into two major landscapes, the Precambrian sandstone Huanchaca plateau (with maximum altitude of 900 m) and the neighboring lowland plains [115]–[116]. The entire region is located in a climatic transition zone [117] and harbors a mosaic of five habitat units that represent distinct ecosystems: upland evergreen forest, deciduous forest, upland cerrado savanna, savanna wetlands, and forest wetlands [78]. The mesa formed by the Serranía de Huanchaca is predominantly covered by savanna, while humid tropical forests are mostly found in the surrounding lowland areas. Seasonally dry forests are not abundant in the region, but occur around rock outcrops and associated with sedimentary soils in localities to the north and south of the mesa [78].

The diversity of habitats found in the Serranía de Huanchaca has been acknowledged as the primary factor accounting for the high levels of species diversity of all groups of organisms surveyed in the area [78], [118]–[122]. However, the region is clearly not sufficiently explored with respect to the biotic diversity it harbors and endemism began to be revealed only recently [78], [121]. The discovery of three conspicuous species of *Tropidurus* endemic to Huanchaca illustrates how insufficient the taxonomic work invested in the area is. It is remarkable that the small area of the Serranía de Huanchaca and its surroundings harbor three endemic lizards species from two distinct clades of the same genus: *T. callathelys* and *T. xanthochilus*, included in the *T. spinulosus* group, and *T. chromatops*, representative of the *T. torquatus* group [4], [9]. Besides demonstrating the occurrence of an extraordinary level of endemism in the area, this discovery highlights its importance for the conservation of phylogenetic diversity within *Tropidurus*, which may be paralleled in other biological groups distributed in Huanchaca.

The three *Tropidurus* species endemic to Huanchaca and surroundings display congruent distributional patterns and high morphological similarity in relation to their phylogenetically closely related species. This pattern was firstly observed by Harvey and Gutberlet [4] who suggested that a single historical event may have been responsible for the evolution of each species pair from common ancestors previously distributed across open formations of south-central South America. Although the phylogenetic position of *T. chromatops* is considered doubtful [9], its distribution associated with rock outcrops surrounded by savannas is a common condition within this clade that includes rupicolous species occupying the savannas of central Brazil. Werneck *et al.* [123] modeled the distribution of the South American savannas from the last interglacial maximum (120,000 yr. BP) and demonstrated that, although separated from the core Cerrado, the savannas of eastern Bolivia remained stable during this period and maintain relationship with the savannas of central Brazil. The progressive erosional cycle (initiated approximately 20 Myr BP) that left the plateau of the Serranía de Huanchaca separated from other mesas in central Brazil [115]–[116] possibly explains the geographic context of isolation of *T. chromatops* and other endemics.

*Tropidurus callathelys* and *T. xanthochilus* are not directly related phylogenetically and display distinct ecologies [4], [9]. The first species inhabits rock outcrops in the Serranía de Huanchaca, while the second is arboricolous and associated with seasonally dry forests [4], [121]. *Tropidurus xanthochilus* and its sister species widely distributed in the Chaco, *T. spinulosus*, were previously suggested to have parapatric distribution where the forests of the Tarvo and Paraguá rivers intergrade with the semideciduous Chiquitano dry forest [4]. However, the closest known populations of *T. spinulosus* is located 350 km south of the type locality of *T. xanthochilus* [4], and despite the distributional data are scarce, the range of these species as currently known still define allopatric distributions [5]. *Tropidurus callathelys* is also allopatric in relation to its sister species, *T. melanopleurus*, which occupies the Andean foothills from northern Argentina to southern Peru [5]. Indeed, the distribution and phylogenetic relationships of both species pairs effectively suggest a single vicariant event as responsible for the origin of the species endemic to Huanchaca. However, no data is currently available to provide an effective test of the temporal congruence between speciation events. To assess the timing of these events is not only essential to properly test the hypothesis of a common diversification history, but also to identify the vicariant processes involved.

### Area Relationships

**Figure 5:**
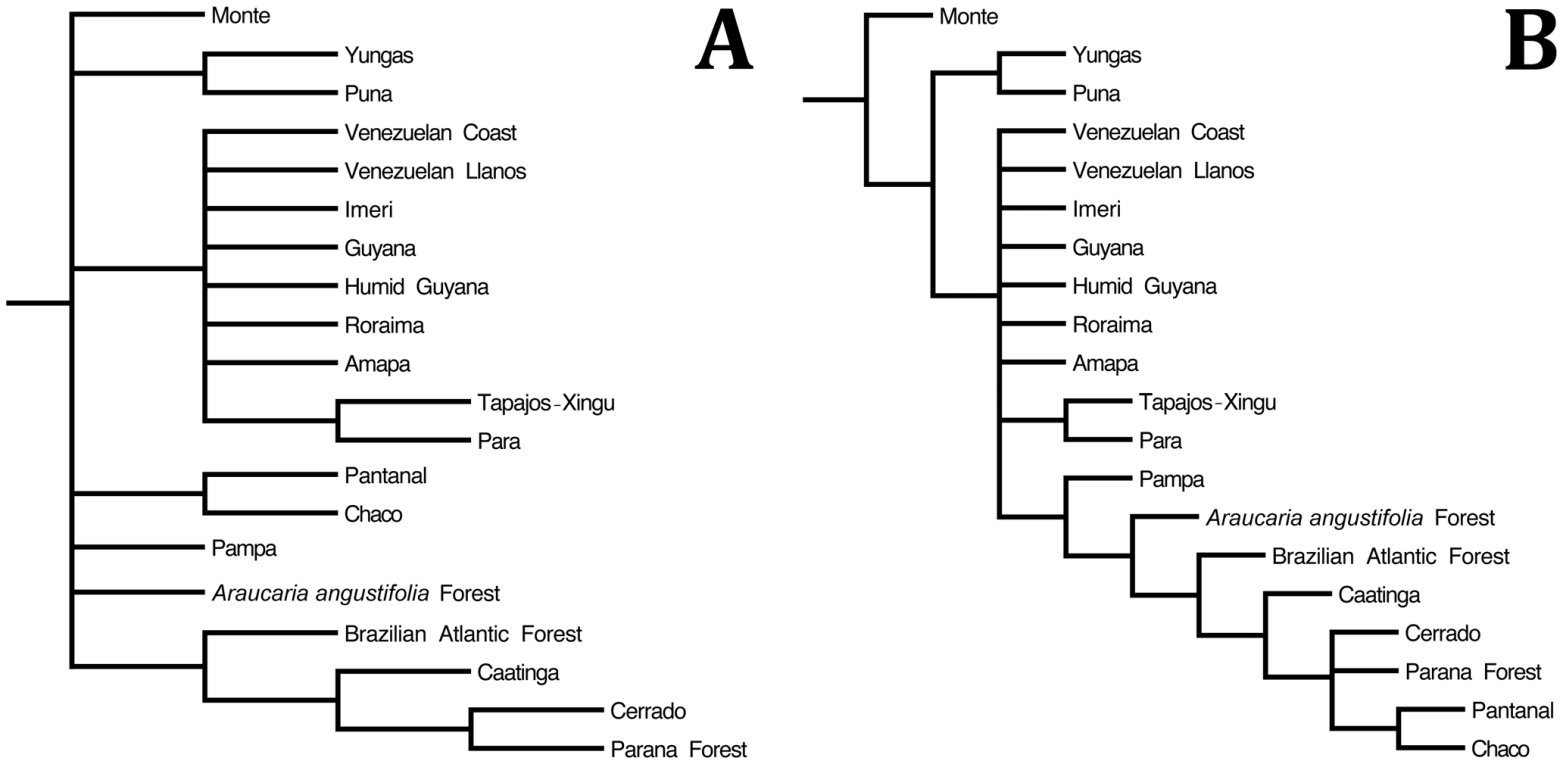
Consensus of the area cladograms generated by (A) Parsimony Analysis of Endemicity (15 trees, L = 38 steps, CI = 0.605, RI = 0.643) and (B) Brooks Parsimony Analysis (2 trees, L = 69 steps, CI = 0.565, RI = 0.694) based on the distribution [**5**] and phylogenetic relationships [**9**] of the species of the lizard genus *Tropidurus*.

PAE identified 15 equally parsimonious trees with length equals to 38 steps, consistency index equals to 0.605, and retention index equals to 0.643. The area cladogram obtained displays nine nodes grouping 20 biogeographic provinces, and shows a basal polytomy grouping seven clades (Fig. 5A). The first was represented by the Monte province. The second grouped the Andean provinces Yungas and Puna. The third grouped provinces located north of the Amazon River (Venezuelan Coast, Venezuelan Llanos, Guyana, Humid Guyana, Roraima) in a polytomy and the clade formed by Tapajós-Xingu and Para. The fourth clade was formed by Chaco and Pantanal. The fifth comprised the Pampa and the sixth the *Araucaria angustifolia* forest. The seventh clade grouped hierarchically the Brazilian Atlantic Forest and provinces of the South American open corridor (Caatinga (Cerrado+Parana Forest).

BPA identified two equally parsimonious trees with length equals to 69 steps, consistency index equals to 0.565, and retention index equals to 0.694. The topology displays 11 nodes grouping 20 biogeographic provinces in 10 clades, and shows significant congruence in relation to that generated by PAE (Fig. 5B, Table 3). The Monte province was recovered as the sister area to all other provinces, which were clustered in two major clades; the first was composed by the Andean provinces Yungas and Puna, and the second comprised provinces located north of the Amazon River (Venezuelan Coast, Venezuelan Llanos, Guyana, Humid Guyana, and Roraima) in a polytomy and Tapajós-Xingu and Para as sister areas. Provinces included in the domains of the Brazilian Atlantic Forest and South American open corridor composed a subclade hierarchically structured. The Pampa was recovered as the most external area, initially separated from the *Araucaria angustifolia* Forest, Brazilian Atlantic Forest, and remaining open provinces. The clade (Caatinga (Cerrado, Parana Forest (Chaco+Pantanal))) was recovered as the most internal subgroup.

**Table 3:** Data matrix of 20 *Tropidurus* species used in the Brooks Parsimony Analysis (BPA) employing the biogeographic provinces of South America as operational geographic units [57]–[58].

Areas	Species																Ancestors																						
	01	02	03	04	05	06	07	08	09	10	11	12	13	14	15	16	17	18	19	20	01	02	03	04	05	06	07	08	09	10	11	12	13	14	15	16	17	18	19
Outgroup	0	0	0	0	0	0	0	0	0	0	0	0	0	0	0	0	0	0	0	0	0	0	0	0	0	0	0	0	0	0	0	0	0	0	0	0	0	0	0
Venezuelan Coast	0	0	0	0	0	0	0	1	0	0	0	0	0	0	0	0	0	0	0	0	0	0	0	0	0	0	1	1	0	0	1	0	1	0	0	1	1	1	1
Venezuelan Llanos	0	0	0	0	0	0	0	1	0	0	0	0	0	0	0	0	0	0	0	0	0	0	0	0	0	0	1	1	0	0	1	0	1	0	0	1	1	1	1
Imeri	0	0	0	0	0	0	0	1	0	0	0	0	0	0	0	0	0	0	0	0	0	0	0	0	0	0	1	1	0	0	1	0	1	0	0	1	1	1	1
Guyana	1	0	0	0	0	0	0	1	0	0	0	0	0	0	0	0	0	0	0	0	0	0	0	0	1	0	1	1	0	0	1	0	1	0	0	1	1	1	1
Humid Guyana	0	0	0	0	0	0	0	1	0	0	0	0	0	0	0	0	0	0	0	0	0	0	0	0	0	0	1	1	0	0	1	0	1	0	0	1	1	1	1
Roraima	0	0	0	0	0	0	0	1	0	0	0	0	0	0	0	0	0	0	0	0	0	0	0	0	0	0	1	1	0	0	1	0	1	0	0	1	1	1	1
Amapa	0	0	0	0	0	0	0	1	0	0	0	0	0	0	0	0	0	0	0	0	0	0	0	0	0	0	1	1	0	0	1	0	1	0	0	1	1	1	1
Tapajos-Xingu	0	0	0	0	0	0	0	1	0	1	0	0	0	0	1	0	0	0	0	0	0	0	0	0	0	1	1	1	0	0	1	0	1	0	0	1	1	1	1
Para	0	0	0	0	0	0	0	1	0	0	0	0	0	0	1	0	1	0	0	0	0	0	0	0	0	1	1	1	0	0	1	0	1	0	0	1	1	1	1
Pantanal	0	1	1	0	0	1	1	0	0	1	1	0	0	0	1	0	0	1	1	1	1	1	1	1	1	1	1	1	0	0	1	1	1	1	1	1	1	1	1
Yungas	0	0	0	0	0	1	0	0	0	0	0	1	0	0	0	0	0	0	0	0	0	1	0	1	1	0	0	0	0	0	0	1	1	0	0	1	1	1	1
Caatinga	0	0	0	1	1	1	0	1	1	0	0	0	1	1	0	1	1	0	0	0	0	0	0	0	0	0	1	1	1	1	1	1	1	1	1	1	1	1	1
Cerrado	0	0	0	0	0	1	1	1	0	1	1	0	1	0	1	0	1	0	1	0	0	0	1	1	1	1	1	1	0	1	1	1	1	1	1	1	1	1	1
Chaco	0	0	0	0	0	1	1	0	0	0	0	1	0	0	0	0	0	1	1	0	1	1	1	1	1	0	1	1	0	0	1	1	1	0	0	1	1	1	1
Pampa	0	0	0	0	0	0	0	0	0	0	0	0	0	0	0	0	0	0	1	0	0	0	0	0	0	0	1	1	0	0	1	0	1	0	0	1	1	1	1
Monte	0	0	0	0	0	0	0	0	0	0	0	0	0	0	0	0	0	1	0	0	1	0	1	1	1	0	0	0	0	0	0	0	0	0	0	0	0	0	1
Brazilian Atlantic Forest	0	0	0	0	0	0	0	1	1	0	1	0	0	0	0	0	1	0	1	0	0	0	0	0	0	0	1	1	0	0	1	0	1	1	1	1	1	1	1
Parana Forest	0	0	0	0	0	1	1	1	0	0	1	0	1	0	0	0	1	0	1	0	0	0	1	1	1	0	1	1	0	1	1	1	1	1	1	1	1	1	1
*Araucaria angustifolia* Forest	0	0	0	0	0	0	0	0	0	0	1	0	0	0	0	0	0	0	1	0	0	0	0	0	0	0	1	1	0	0	1	0	1	1	1	1	1	1	1
Puna	0	0	0	0	0	1	0	0	0	0	0	1	0	0	0	0	0	0	0	0	0	1	0	1	1	0	0	0	0	0	0	1	1	0	0	1	1	1	1

Absence of taxon codified as “0”, presence codified as “1”. Taxa: (1) *T. bogerti*, (2) *T. callathelys*, (3) *T. chromatops*, (4) *T. cocorobensis*, (5) *T. erythrocephalus*, (6) *T. etheridgei*, (7) *T. guarani*, (8) *T. hispidus*, (9) *T. hygomi*, (10) *T. insulanus*, (11) *T. itambere*, (12) *T. melanopleurus*, (13) *T. montanus*, (14) *T. mucujensis*, (15) *T. oreadicus*, (16) *T. psammonastes*, (17) *T. semitaeniatus*, (18) *T. spinulosus*, (19) *T. torquatus*, (20) *T. xanthochilus*. Note: *T. helenae*, *T. jaguaribanus, and T. pinima* (from Caatinga province) were not included since these species were absent from the phylogenetic hypothesis of Frost *et al.* [9], used for implementation of BPA. Hypothetical ancestors are represented in Fig. 1.

The employment of different analytical methods and operational geographic units resulted in area cladograms that were congruent with previously published hypotheses, recovering a close relationship between the Atlantic Forest and areas of the South American open corridor (Caatinga, Cerrado, and Chaco) [30], [32], [88]. The congruence of the results generated from different databases significantly increases the reliability of the patterns documented. However, the possibly composite nature of the Atlantic Forest must be considered [30], [124]–[125]. The Brazilian Atlantic Forest exhibits the largest latitudinal extension among the provinces included in this study and significant shifts in faunal composition that define nested areas of endemism along its wide range [125]–[126]. In addition, biotic exchanges with the Amazon rainforest during pulses of forest expansion and retraction driven by climatic cycles resulted in a complex biogeographic history and intricate patterns of area relationships [124]–[127]. We acknowledge that a detailed investigation of the biogeographic history of the Brazilian Atlantic Forest is beyond the limits of the database analyzed since *Tropidurus* is not effectively associated with forested environments and occupies exclusively granitic inselbegs, open formation enclaves, and coastal white sand dunes along the province [1], [5].

The Pampa was positioned by BPA as the most basal area of the clade that comprises the Brazilian Atlantic Forest, Caatinga, Cerrado, Parana Forest, Pantanal, and Chaco. This area has been linked to Patagonia and Andes [30], but is considered part of the South American open corridor [128]. Porzecanski and Cracraft [30] highlighted the physiognomic heterogeneity presented by the Pampa–which carries a mosaic of physiognomies of Monte, Chaco, and Atlantic Forest–and proposed the structural diversity of this area as a possible factor responsible for guaranteeing “hospitality” to dispersal of organisms coming from adjacent provinces. The incongruent relationship patterns observed for the Pampa indicate either mixed interrelationships resulting from multiple vicariance histories or the effect of large numbers of cases of dispersion [30]. The only representative of *Tropidurus* found in the Pampa is *T. torquatus*, a species phylogenetically derived [9], ecologically generalist and widely distributed [1], [5]. The occurrence of this species in the Pampa does not provide factual information about historical relationships of that province, and possibly results from its great ecological plasticity and dispersive capacity.

Our results corroborate a close relationship among the Caatinga, Cerrado and Chaco, a pattern noted in previous studies employing different groups of organisms [30], [129]–[130]. The BPA cladogram supports the separation of the Atlantic Forest from the South American open corridor and subsequent split between Caatinga and (Cerrado, Parana Forest+(Pantanal+Chaco)). Cerrado (including the Pantanal) and Chaco have been recurrently recovered as sister areas and this relationship attributed to different putative vicariant events (review in Ref. [33]): (1) establishment of humid forest corridors connecting Amazon and Atlantic Forest and conversely segregating Caatinga from Chaco+Cerrado [13], [131]–[132]; (2) the uplift of the Brazilian Plateau along the Espinhaço range, Serra do Mar and Serra da Mantiqueira (Late Pliocene-Early Pleistocene, 4-2 Myr BP), and (3) the subsidence of the Chaco and Pantanal due to the Andean uplift [30], [32]. However, Werneck [33] highlighted that the close relationship between the Cerrado and Chaco should be considered with caution, since there is evidence that the Chaco is more directly related to dry formations of southern South America [75]. Indeed, the distribution models produced by Werneck *et al.* [123] indicate that the Cerrado’s biogeographical counterparts are not Chaco and Caatinga but rather the disjunct savannas of the Guyana shield plateau (Gran Sabana and Llanos). Nevertheless, the models produced are limited to the last interglacial maximum (120,000 years BP) and do not represent the last word regarding the origin and relationships between open areas of South America. Our results in general corroborate a consistent pattern recovered by several studies comprising different groups of organism (review in Ref. [130]), but we highlight that the effective assessment of the history of the areas analyzed must rely on both the spatial and temporal congruence of the diversification events involving these groups to properly refute misleading interpretations of putative vicariant scenarios.

Aiming to avoid speculative scenarios, we made the decision to not associate the patterns of area relationships recovered with specific vicariant events. No temporal framework is currently established with respect to the diversification of *Tropidurus.* Hence, it is premature to relate cladogenetic events with specific time periods or putative geographic barriers resulting from geological, climatological or landscape changes occurred in South America. Nevertheless, the recent recognition of a clear pattern of distribution of endemism within the Cerrado, marked by the occurrence of distinct faunas associated with plateaus and peripheral depressions [133]–[134], demonstrates that the uplift of the Brazilian Central Plateau impacted decisively the biogeographic history of numerous vertebrate groups in South America [32], [129]–[130], [133]–[134]. In addition, the identification of a diversification history mostly defined in the Tertiary for other squamate groups with similar distributions and ecological requirements [96], [135] suggests that *Tropidurus* is not an exception. The lack of an appropriate taxonomic resolution of the species complexes currently represented by widely distributed forms, the need for a robust all-inclusive phylogenetic hypothesis, and the absence of temporal references in relation to the diversification of the distinct clades within *Tropidurus* are recognized as limiting factors hampering the understanding of the biogeographic history of the group. We emphasize that these three important aspects concerning the evolutionary history of these lizards should be prioritized in future investigations.

## References

[1] Rodrigues MT (1987) Sistemática, ecologia e zoogeografia dos *Tropidurus* do grupo torquatus ao sul do Rio Amazonas (Sauria, Iguanidae). Arq Zool 31: 105–230.

[2] Rodrigues MT (1988) Distribution of lizards of the genus *Tropidurus* in Brazil (Sauria, Iguanidae). In: Vanzolini PE, Heyer WR, editors. Proceedings of a workshop on neotropical distribution patterns. Rio de Janeiro: Academia Brasileira de Ciências. 305–315.

[3] Ávila-Pires TCS (1995) Lizards of Brazilian Amazonia (Reptilia: Squamata). Zool Verhand 299: 1–706.

[4] Harvey MB, Gutberlet RL Jr (1998) Lizards of the genus *Tropidurus* (Iguania: Tropiduridae) from the Serrania de Huanchaca, Bolivia: new species, natural history, and a key to the genus. Herpetologica 54: 493–520.

[5] Carvalho ALG (2013) On the distribution and conservation of the South American lizard genus *Tropidurus* Wied-Neuwied, 1825 (Squamata: Tropiduridae). Zootaxa: In press. 10.11646/zootaxa.3640.1.3 26000403

[6] Frost DR (1992) Phylogenetic analysis and taxonomy of the *Tropidurus* group of lizards (Iguania: Tropidurudae). Am Mus Novit 3033: 1–68.

[7] Frost DR, Crafts HM, Fitzgerald LA, Titus TA (1998) Geographic variation, species recognition, and molecular evolution of cytochrome oxidase I in the *Tropidurus spinulosus* complex (Iguania: Tropiduridae). Copeia: 839–851.

[8] Harvey MB, Gutberlet RL Jr (2000) A phylogenetic analysis of the tropidurine lizards (Squamata: Tropiduridae), including new characters of squamation and epidermal microstructure. Biol J Linn Soc 128: 189–233.

[9] Frost DR, Rodrigues MT, Grant T, Titus TA (2001) Phylogenetics of the lizard genus *Tropidurus* (Squamata: Tropiduridae: Tropidurinae): direct optimization, descriptive efficiency, and sensitivity analysis of congruence between molecular data and morphology. Mol Phyl Evol 21: 352–371. 10.1006/mpev.2001.1015 11741379

[10] Haffer J (1969) Speciation in Amazonian Forest Birds. Science 165: 131–137. 17834730 10.1126/science.165.3889.131

[11] Vanzolini PE, Williams EE (1981) The vanishing refuge: a mechanism for ecogeographic speciation. Pap Avul Zool 34: 251–255.

[12] Prance GT (1973) Phytogeographic support for the theory of Pleistocene forest refuges in the Amazon Basin, based on evidence from distribution patterns in Caryocaraceae, Chrysobalanaceae, Dichapetalaceae and Lecythidaceae. Acta Amazon 3: 5–25.

[13] Bigarella JJ, Lima DA, Richs PJ (1975) Considerações a respeito das mudanças paleoambientais na distribuição de algumas espécies vegetais e animais do Brasil. An Acad Bras Cienc 47 (Supl.): 411–464.

[14] Brown KS, Ab’Saber NA (1979) Ice-age forest refuges and evolution in Neotropics: correlation of paleoclimatological, geomorphological and pedological data with biological endemism. Paleoclimas 5: 1–30.

[15] Haffer J, Prance GT (2001) Climatic forcing of evolution in Amazonia during the Cenozoic: On the refuge theory of biotic differentiation. Amazoniana 16: 579–608.

[16] Knapp S, Mallet J (2003) Refuting Refugia? Science 300 (5616): 71–72. 10.1126/science.1083007 12677050

[17] Bush MB, Oliveira PE (2006) The rise and fall of the Refugial Hypothesis of Amazonian speciation: a paleoecological perspective. Biota Neotrop 6: 1.

[18] Heyer WR, Maxson LR (1983) Relationships, zoogeography, and speciation mechanisms of frogs of the genus *Cycloramphus* (Amphibia, Leptodactylidae). Arq Zool 30: 341–73.

[19] Vanzolini PE, Heyer WR (1988) Proceedings of a workshop on Neotropical distribution patterns. Rio de Janeiro: Academia Brasileira de Ciências. 488 p.

[20] Gainsbury AM, Colli GR (2003) Lizard assemblages from natural cerrado enclaves in southwestern Amazonia: The role of stochastic extinctions and isolation. Biotropica 35: 503–519.

[21] De Vivo M, Carmignotto AP (2004) Holocene vegetation change and the mammal faunas of South America and Africa. J Biogeogr 31: 943–957.

[22] Borges-Nojosa DM, Caramaschi U (2005) Composição e análise comparativa da diversidade e das afinidades biogeográficas dos lagartos e anfisbenídeos (Squamata) dos brejos nordestinos. In: Araújo, FS, Rodal, MJN, Barbosa, MRV, editors. Análise das variações da biodiversidade do bioma Caatinga: suporte a estratégias regionais de conservação. Brasília: Ministério do Meio Ambiente. 463–512.

[23] Werneck FP, Colli GR (2006) The lizard assemblage from seasonally dry tropical forest enclaves in the Cerrado biome, Brazil, and its association with the Pleistocenic Arc. J Biogeogr 33: 1983–1992.

[24] Almeida FC, Bonvicino CR, Cordeiro-Estrela P (2007) Phylogeny and temporal diversification of *Calomys* (Rodentia, Sigmodontinae): implications for the biogeography of an endemic genus of the open/dry biomes of South America. Mol Phyl Evol 42: 449–466. 10.1016/j.ympev.2006.07.005 16971145

[25] Lundberg JG, Marshall LG, Guerrero J, Horton B, Malabarba MCSL, et al.. (1998) The stage for neotropical fish diversification: a history of tropical South American rivers. In: Malabarba LR, Reis RE, Vari RP, Lucena ZM, Lucena CAS, editors. Phylogeny and classification of Neotropical fishes. Porto Alegre: Editora PUC Rio Grande do Sul. 13–48.

[26] Cortés-Ortiz L, Bermingham E, Rico C, Rodríguez-Luna E, Sampaio I, et al (2003) Molecular systematics and biogeography of the Neotropical monkey genus *Alouatta* . Mol Phyl Evol 26: 64–81. 10.1016/s1055-7903(02)00308-1 12470939

[27] Rull V (2008) Speciation timing and Neotropical biodiversity: the Tertiary-Quaternary debate in the light of molecular phylogenetic evidence. Mol Ecol 17: 2722–2729. 18494610 10.1111/j.1365-294X.2008.03789.x

[28] Antonelli A, Quijada-Mascareñas A, Crawford AJ, Bates JM, Velazco PM, et al.. (2010) Molecular studies and phylogeography of Amazonian tetrapods and their relation to geological and climatic models. In: Hoorn C, Wesselingh FP, editors. Amazonia, landscapes and species evolution: a look into the past. 1st ed. Oxford: Blackwell. 386–404.

[29] Cracraft J (1985) Historical biogeography and the patterns of differentiation within the South American avifauna: areas of endemism. Ornithol Monogr 36: 49–84.

[30] Porzecanski AL, Cracraft J (2005) Cladistic analysis of distributions and endemism (CADE): using raw distributions of birds to unravel the biogeography of the South American arid lands. J Biogeogr 32: 261–275.

[31] Haffer J (1985) Avian zoogeography in the Neotropical lowlands. Ornithol Monogr 36: 113–146.

[32] Colli GR (2005) As origens e a diversificação da herpetofauna do Cerrado. In: Scariot A, Souza-Silva JCD, Felfili JM, editors. Cerrado: Ecologia, Biodiversidade e Conservação. Brasília: Ministério do Meio Ambiente. 247–264.

[33] Werneck FP (2011) The diversification of eastern South American open vegetation biomes: historical biogeography and perspectives. Quaternary Sci Rev 30: 1630–1648.

[34] Google Inc. (2011) Google Earth (Version 6.1.0.5001). Available: http://www.google.com/earth/index.html. Accessed 2012 Apr 2.

[35] ESRI (2011) ArcGIS Desktop: Release 10. Environmental Systems Research Institute, Redlands.

[36] Rosen BR (1988) From fossils to earth history: applied historical biogeography. In: Myers AA, Giller PS, editors. Analytical Biogeography - An integrated approach to the study of animal and plant distributions. London: Madras, Chapman and Hall. 437–481.

[37] Rosen BR, Smith AB (1988) Tectonics from fossils? Analysis of reef-coral and sea-urchin distributions from late Cretaceous to Recent, using a new method. In: Audley-Charles MG, Hallam A, editors. Gondwana and Tethys. London, Geological Society of London. Special publication no. 37: 275–306.

[38] Morrone JJ, Crisci JV (1995) Historical biogeography: introduction to methods. Annu Rev Ecol Evol Syst 26: 373–401.

[39] Crisci JV, Katinas L, Posadas P. (2000) Introducción a la teoria y práctica de la biogeografía histórica. Buenos Aires, Sociedad Argentina de Botánica. 169 p.

[40] Zandee M, Roos MC (1987) Component-compatibility in historical biogeography. Cladistics 3: 305–332. 10.1111/j.1096-0031.1987.tb00896.x 34949059

[41] Brooks DR, Van Veller MGP (2003) Critique of parsimony analysis of endemicity as a method of historical biogeography. J Biogeogr 30: 819–825.

[42] Santos CMD (2005) Parsimony analysis of endemicity: time for a epitaph? J Biogeogr 32: 1284–1286.

[43] Nihei SS (2006) Misconceptions about parsimony analysis of endemicity. J Biogeogr 33: 2099–2106.

[44] Garzón-Orduña IJ, Miranda-Esquivel DR, Donato M (2007) Parsimony analysis of endemicity describes but does not explain: an illustrated critique. J Biogeogr 35: 903–913.

[45] Morrone JJ (1994) On the identification of areas of endemism. Syst Biol 43: 438–441.

[46] Platnick NI (1991) On areas of endemism. Austral Syst Bot 4: not numbered.

[47] Harold AS, Mooi RD (1994) Areas of endemism: definition and recognition criteria. Syst Biol 43: 261–266.

[48] Nihei SS (2008) Dynamic endemism and ‘general’ biogeographic patterns. Biogeografia 3: 2–6.

[49] Cracraft J (1982) Geographic differenciation, cladistics, and vicariance biogeography: reconstructing the tempo and mode of evolution. Amer Zool 22: 411–424.

[50] Cracraft J (1983) Cladistic analysis and vicariance biogeography. Amer Sci 71: 273–281.

[51] Cracraft J (1986) Origin and evolution of continental biotas: speciation and historical congruence within the Australian avifauna. Evolution 40: 977–996. 28556211 10.1111/j.1558-5646.1986.tb00566.x

[52] Cracraft J (1991) Patterns of diversification within continental biotas: hierarchical congruence among the areas of endemism of Australian vertebrates. Austral Syst Bot 4: 211–227.

[53] Cracraft J (1994) Species diversity, biogeography, and the evolution of biotas. Amer Zool 34: 33–47.

[54] Hausdorf B (2002) Units in Biogeography. Syst Biol 51: 648–652. 12228006 10.1080/10635150290102320

[55] Rosen BR (1978) Vicariant patterns and historical explanation in Biogeography. Syst Zool 27: 159–188.

[56] Nelson G, Platnick NI (1981) Systematics and Biogeography - Cladistics and Vicariance. New York, Columbia University Press. 567 p.

[57] Morrone JJ (2004) Panbiogeografia, componentes bióticos y zonas de transición. Rev Bras Entomol 48: 149–162.

[58] Morrone JJ (2006) Biogeographic areas and transition zones of Latin America and the Caribbean Islands based on panbiogeographic and cladistic analyses of the entomofauna. Annu Rev Entomol 51: 467–494. 16332220 10.1146/annurev.ento.50.071803.130447

[59] Margush T, McMorris FR (1981) Consensus n-trees. B Math Biol 43: 239–244.

[60] Brooks DR (1981) Hennig’s parasitological method: a proposed solution. Syst Zool 30: 229–249.

[61] Brooks DR (1985) Historical ecology: a new approch to studying the evolution of ecological associations. Ann Missouri Bot Gard 72: 660–680.

[62] Brooks DR (1988) Macroevolutionary comparisons of host and parasite phylogenies. Annu Rev Ecol Evol Syst 19: 235–259.

[63] Wiley EO (1988a) Vicariance biogeography. Annu Rev Ecol Syst 19: 513–542.

[64] Wiley EO (1988b) Parsimony analysis and vicariance biogeography. Syst Zool 37: 271–290.

[65] Brooks DR (1990) Parsimony analysis in historical biogeography and coevolution: methodological and theoretical update. Syst Zool 39: 14–30.

[66] Kluge AG (1988) Parsimony in vicariance biogeography: a quantitative method and a Greater Antillean example. Syst Zool 37: 315–328.

[67] Brooks DR, Van Veller MGP, McLennan DA (2001) How to do BPA, really. J Biogeogr 28: 345–358.

[68] McLennan DA, Brooks DR (2002) Complex histories of speciation and dispersion in communities: a re-analysis of some Australian bird data using BPA. J Biogeogr 29: 1055–1066.

[69] Goloboff PA, Farris S, Nixon K (2000) TNT (Tree analysis using New Technology) (BETA). Published by the authors, Tucumán, Argentina.

[70] Swofford DL (2001) Phylogenetic Analysis Using Parsimony (PAUP), version 4.0.10b. Washington, D.C.

[71] Goloboff PA (1999) Analyzing large data sets in reasonable times: solutions for composite optima. Cladistics 15: 415–428. 10.1111/j.1096-0031.1999.tb00278.x 34902941

[72] Nixon KC (1999) The parsimony ratchet, a new method for rapid parsimony analysis. Cladistics 15: 407–414. 10.1111/j.1096-0031.1999.tb00277.x 34902938

[73] Kohlsdorf T, Garland T Jr, Navas CA (2001) Limb and tail lengths in relation to substrate usage in Tropidurus lizards. J Morphol 248: 151–164. 11304746 10.1002/jmor.1026

[74] Grizante MB, Navas CA, Garland T Jr, Kohlsdorf T (2010) Morphological evolution in Tropidurinae lizards: an integrated view along a continuum of ecological settings. J Evol Biol 23: 98–111. 19895656 10.1111/j.1420-9101.2009.01868.x

[75] Prado DE, Gibbs PE (1993) Patterns of species distributions in the dray seasonal forests of South America. Ann Missouri Bot Gard 80: 902–927.

[76] Parado DE (2000) Seasonally dry forests of tropical South America: from forgotten ecosystems to a new phytogeographic unit. Edinburgh J Bot 57: 437–461.

[77] Werneck FP, Costa GC, Colli GR, Prado DE, Sites JW Jr (2011) Revisiting the historical distribution of seasonally dry tropical forests: new insights based on palaeodistribution modelling and palynological evidence. Global Ecol Biogeogr 20: 272–288.

[78] Killeen TJ (1998) Vegetación y Flora del Parque Nacional Noel Kempff Mercado. In: Killeen TJ, Schulenberg TS, editors. A biological assessment of Parque Nacional Noel Kempff Mercado, Bolivia. RAP Working Papers 10, Washington. Conservation International. 61–85.

[79] Morrone JJ, Escalante T (2002) Parsimony analysis of endemicity (PAE) of Mexican terrestrial mammals at different area units: when size matters. J Biogeogr 29: 1095–1104.

[80] Sick H (1965) A fauna do Cerrado. Arq Zool 12: 71–93.

[81] Vanzolini PE (1974) Ecological and geographical distribution of lizards in Pernambuco, northeastern Brasil (Sauria). Pap Avul Zool 28: 61–90.

[82] Vanzolini PE (1976) On the lizards of a Cerrado-Caatinga contact: evolutionary and zoogeographical implications (Sauria). Pap Avul Zool 29: 111–119.

[83] Mares MA, Willig MR, Streilein KE, Lacher TE Jr (1981) The mammals of northeastern Brazil: a preliminary assessment. Ann Carnegie Mus 50: 80–137.

[84] Mares MA, Willig MR, Streilein KE, Lacher TE Jr (1985) The Brazilian Caatinga in South American zoogeography: tropical mammals in a dry region. J Biogeogr 12: 57–69.

[85] Rodrigues MT (1996) Lizards, snakes, and amphisbaenians from the “Quaternary” sand dunes of the middle rio São Francisco, Bahia, Brazil. J Herpetol 30: 513–523.

[86] Silva JMC, Tabarelli M, Fonseca MT, Lins LV (2004) Biodiversidade da Caatinga: áreas e ações prioritárias para a conservação. Brasília: Ministério do Meio Ambiente, Universidade Federal de Pernambuco. 382 p.

[87] Juncá FA (2005) Anfíbios e répteis. In: Juncá FA, Funch L, Rocha W, editors. Biodiversidade e conservação da Chapada Diamantina. Brasília: Ministério do Meio Ambiente. 337–356.

[88] Roig-Juñent S, Domínguez MC, Flores GE, Mattoni C (2006) Biogeographic history of South American arid lands: a view from its arthropods using TASS analysis. J Arid Environ 66: 404–420.

[89] Prado DE (2003) As caatingas da América do Sul. In: Leal, LR, Tabarelli, M, Silva, JMC, editors. Ecologia e conservação da caatigna. Recife, Editora Universitária da UFPE. 3–73.

[90] Velloso AL, Sampaio EVSB, Pareyn FGC (2002) Ecorregiões propostas para o Bioma caatinga. Recife: Associação Plantas do Nordeste, Instituto de Conservação Ambiental, The Nature Conservancy do Brasil. 76 p.

[91] Queiroz LP (2006) The Brazilian Caatinga: phytogeographical patterns inferred from distribution data of the Leguminosae. In: Pennington RT, Lewis GP, Ratter JA, editors. Neotropical savannas and seasonally dry forests: plant diversity, biogeography and conservation. Boca Raton, London, New York, CRC Press Taylor & Brancis Group. 113–149.

[92] Rodrigues MT (2003) Herpetofauna da Caatinga. In: Leal LR, Tabarelli M, Silva JMC, editors. Ecologia e conservação da Caatinga. Recife, Editora Universidade da UFPE. 181–236.

[93] Rodrigues MT, Kasahara S, Yonenaga-Yassuda Y (1988) *Tropidurus psammonastes*: uma nova espécie do grupo *torquatus* com notas sobre seu cariótipo e distribuição (Sauria, Iguanidae). Pap Avul Zool 36: 307–313.

[94] Rodrigues MT (1986) Um novo *Tropidurus* com crista dorsal do Brasil, com comentários sobre suas relações, distribuição e origem (Sauria, Iguanidae). Pap Avul Zool 36: 171–179.

[95] Rodrigues MTU (1995) Filogenia e história geográfica de uma radiação de lagartos microteídeos (Sauria, Teiioidea, Gymnophthalmidae). Tese de livre docência. Universidade de São Paulo. 92 p.

[96] Passoni JC, Benozzati ML, Rodrigues MT (2008) Phylogeny, species limit, and biogeography of the Brazilian lizards of the genus *Eurolophosaurus* (Squamata: Tropiduridae) as inferred from mitocondrial DNA sequences. Mol Phylogenet Evol 46: 403–414. 18082430 10.1016/j.ympev.2007.10.022

[97] Siedchlag AC, Benozzati ML, Passoni JC, Rodrigues MT (2010) Genetic structure, phylogeny, and biogeography of Brazilian eyelid-less lizards of genera *Calyptommatus* and *Nothobachia* (Squamata, Gymnophthalmidae) as inferred from mitochondrial DNA sequences. Mol Phyl Evol 56: 622–630. 10.1016/j.ympev.2010.04.027 20434569

[98] Rapini1 A, Ribeiro PL, Lambert S, Pirani JR (2008) A flora dos campos rupestres da Cadeia do Espinhaço. Megadiversidade 4: 16–24.

[99] Giulietti AM, Pirani JR (1988) Patterns of geographical distribution of some plant species from Espinhaço range, Minas Gerais and Bahia, Brazil. In: Vanzolini PE, Heyer WR, editors. Proceedings of a workshop on Neotropical distribution patterns. Riode Janeiro, Academia Brasileira de Ciências. 39–69.

[100] Giulietti AM, Pirani JR, Harley RM (1997) Espinhaço range region. Eastern Brazil. In: Davis SD, Heywood VH, Herrera-MacBryde O, Villa-Lobos J, Hamilton AC, editors. Centres of plant diversity. A guide and strategies for the conservation, Vol. 3. The Americas. Cambridge, WWF/IUCN. 397–404.

[101] Leite FSF, Juncá FA, Eterovick PC (2008) Status do conhecimento, endemismo e conservação de anfíbios anuros da Cadeia do Espinhaço, Brasil. Megadiversidade 4: 158–176.

[102] Vasconcelos MF, Lopes LE, Machado MG, Rodrigues M (2008) As aves dos campos rupestres da Cadeia do Espinhaço: diversidade, endemismo e conservação. Megadiversidade 4: 197–217.

[103] Alves CBM, Leal CG, Brito MFG, Santos ACA (2008) Biodiversidade e conservação de peixes do Complexo do Espinhaço. Megadiversidade 4: 177–196.

[104] Vasconcelos MF (2008) Mountaintop endemism in eastern Brazil: why some bird species from campos rupestres of the Espinhaço Range are not endemic to the Carrado region? Rev Bras Ornitol 16: 348–362.

[105] Echternacht L, Trovóa M, Oliveira CT, Pirani JR (2011) Areas ofendemism in the Espinhaço Range in Minas Gerais, Brazil. Flora - Morphology, Distribution, Functional Ecology of Plants 206: 782–791.

[106] Vitt LJ (1981) Lizard reproduction: habitat specificit and constraints on relative clutch mass. Amer Nat 117: 506–514.

[107] Vanzolini PE, Ramos-Costa AMM, Vitt LJ (1980) Répteis das Caatingas. Rio de Janeiro: 161 p.

[108] Vitt LJ, Price HJ (1982) Ecological and evolutionary determinants of relative clutch mass. Am Nat 117: 506–514.

[109] Rodrigues MT (1984) Sobre *Platynotus* Wagler, 1830, pré-ocupado, substituído por *Tapinurus* Amaral, 1933, com a descrição de uma nova espécie (Sauria, Iguanidae). Pap Avul Zool 35: 367–373.

[110] Manzani PR, Abe A (1990) A new species of *Tapinurus* from the Caatinga of Piaui, Northeastern Brazil (Squamata: Tropiduridae). Herpetol J 46: 462–467.

[111] Passos DC, Lima DC, Borges-Nojosa DM (2011) A new species of *Tropidurus* (Squamata, Tropiduridae) of the *semitaeniatus* group from a semiarid area in Northeastern Brazil. Zootaxa 2930: 60–68.

[112] Jablonski D, Roy K, Valentine JW (2006) Out of the tropics: evolutionary dynamics of the latitudinal diversity gradient. Science 314: 102–106. 17023653 10.1126/science.1130880

[113] Graham CH, Moritz C, Williams SE (2006) Habitat history improves prediction of biodiversity in rainforest fauna. Proc Nat Acad Sci USA 103: 632–636. 16407139 10.1073/pnas.0505754103 PMC1334636

[114] Carnaval AC, Moritz C (2008) Historical climate modelling predicts patterns of current biodiversity in the Brazilian Atlantic Forest. J Biogeogr 35: 1187–1201.

[115] Litherland M, Power G (1989) The geological and geomorphological evolution of the Serranía Huanchaca, eastern Bolivia: The legendary “Lost World.”. J S Am Earth Sci 2: 1–17.

[116] Killeen TJ (1998) Introduction. Geomorphology of the Huanchaca Plateau and surrounding areas. In: Killeen TJ, Schulenberg TS, editors. A biological assessment of Parque Nacional Noel Kempff Mercado, Bolivia. RAP Working Papers 10. Washington, Conservation International. 43–45.

[117] Killeen TJ (1998) Introduction. Climate and paleoclimate. In: Killeen TJ, Schulenberg TS, editors. A biological assessment of Parque Nacional Noel Kempff Mercado, Bolivia. RAP Working Papers 10. Washington, Conservation International. 48–51.

[118] Bates JM, Stotz DF, Schulenberg TS (1998) Avifauna of Parque Nacional Noel Kempff Mercado. In: Killeen TJ, Schulenberg TS, editors. A biological assessment of Parque Nacional Noel Kempff Mercado, Bolivia. RAP Working Papers 10. Washington, Conservation International. 112–119.

[119] Emmans LH (1998) Mammal fauna of Parque Nacional Noel Kempff Mercado. In: Killeen TJ, Schulenberg TS, editors. A biological assessment of Parque Nacional Noel Kempff Mercado, Bolivia. RAP Working Papers 10. Washington, Conservation International. 129–135.

[120] Forsyth AB, Spector S, Gill B, Guerra F, Ayzama S (1998) Dung beetles (Coleoptera: Scarabaeidae: Scarabaeinae) of Parque Nacional Noel Kempff Mercado. In: Killeen TJ, Schulenberg TS, editors. A biological assessment of Parque Nacional Noel Kempff Mercado, Bolivia. RAP Working Papers 10. Washington, Conservation International. 181–190.

[121] Harvey MB (1998) Reptiles and amphibians of Parque Nacional Noel Kempff Mercado. In: Killeen TJ, Schulenberg TS, editors. A biological assessment of Parque Nacional Noel Kempff Mercado, Bolivia. RAP Working Papers 10. Washington, Conservation International. 144–153.

[122] Sarmiento J (1998) Ichthyology of Parque Nacional Noel Kempff Mercado. In: Killeen TJ, Schulenberg TS, editors. A biological assessment of Parque Nacional Noel Kempff Mercado, Bolivia. RAP Working Papers 10. Washington, Conservation International. 167–173.

[123] Werneck FP, Nogueira C, Colli GR, Sites JW Jr, Costa GC (2012) Climatic stability in the Brazilian Cerrado: implications for biogeographical connections of South American savannas, species richness and conservation in a biodiversity hotspot. J Biogeogr 39: 1695–1706.

[124] Santos AMM, Cavalcanti DR, Silva JMC, Tabarelli M (2007) Biogeographical relationships among tropical forests in north-eastern Brazil. J Biogeogr 34: 1284–1286.

[125] Da Silva MB, Pinto-da-Rocha R (2010) História biogeográfica da Mata Atlântica: opiliões (Arachinida) como modelo para sua inferência. In: Carvalho, CJB, Almeida, EAB, editors. Biogeografia da América do Sul – padrões e processos. São Paulo: Editora Roca. 221–238.

[126] Costa LP, Leite YLR, Fonseca GAB, Fonseca MT (2000) Biogeography of South American forest mammals: endemism and diversity in the Atlantic Forest. Biotropica 32: 872–881.

[127] Caetano S, Prado D, Pennington RT, Beck S, Oliveira-Filho A, et al (2008) The history of Seasonally Dry Tropical Forests in eastern South America: inferences from the genetic structure of the tree Astronium urundeuva (Anacardiacae). Mol Ecol 17: 3147–3159. 18522691 10.1111/j.1365-294X.2008.03817.x

[128] Morrone JJ (2000) What is the Chacoan subregion? Neotropica 46: 51–58.

[129] Silva JMC (1995) Biogeographic analysis of the South American Cerrado avifauna. Steenstrupia 21: 49–67.

[130] Zanella FCV (2010) Evolução da biota da diagonal de formações secas da América do Sul. In: Carvalho CJB, Almeida EAB, editors. Biogeografia da América do Sul – padrões e processos. São Paulo: Editora Roca. 198–220.

[131] Andrade-Lima D (1982) Present-day forest refuges in northeastern Brazil. In: Prance GT, editor. Biological diversification in the Tropics. New York, Columbia University Press. 245–251.

[132] Costa LP (2003) The historical bridge between the Amazon and the Atlantic Forest of Brazil: a study of molecular phylogeography with small mammals. J Biogeogr 30: 71–86.

[133] Nogueira C (2006) Diversidade e padrões de distribuição da fauna de lagartos do Cerrado. Unpublished PhD Thesis, Departamento de Ecologia, Universidade de São Paulo. 297 p.

[134] Nogueira C, Ribeiro S, Costa GC, Colli GR (2011) Vicariance and endemism in a Neotropical savanna hotspot: distribution patterns of Cerrado squamate reptiles. J Biogeogr 38: 1907–1922.

[135] Werneck FP, Gamble T, Colli GR, Rodrigues MT, Sites JW Jr (2012) Deep diversification and long-term persistence in the South American ‘dry diagonal’: Integrating continent-wide phylogeography and distribution modeling in geckos. Evolution 66: 3014–3034. 23025595 10.1111/j.1558-5646.2012.01682.x

